# Novel approximate adaptive carry lookahead adder for error resilient applications with generic method for error analysis

**DOI:** 10.1038/s41598-025-03865-0

**Published:** 2025-06-01

**Authors:** Viraj Joshi, Pravin Mane

**Affiliations:** Department of Electrical and Electronics Engineering, Birla Institute of Technology and Science, Pilani, K K Birla Goa Campus, Zuarinagar, Sancoale, Goa 403726 India

**Keywords:** Approximate computing (AC), Error rate (ER), Acceptance probability (AP), Reconfigurable approximate carry lookahead adder (RAP-CLA), Gray cell, Black cell, Engineering, Mathematics and computing

## Abstract

The paper presents a novel architecture of approximate adder and a novel generic error analysis method. The proposed architecture judiciously make use of time axis based parallelism of components to improve delay and simultaneously improvement in error parameters due to adaptive increase in group-size of carry generating blocks. The synthesis of architectures for same bit length has shown area, power, and delay improvement by 4.91%, 5.59%, 14.92% respectively with respect to state of the art architectures based on truncation of carry chain when synthesized under same constraints and same operating conditions. In comparison to ETA-I, ETA-II and GeAr, proposed method has shown improvement in delay by 9%, 17.9% and 21.3% respectively. Error analysis is done for proposed adder using random probabilistic method and generic analysis method. The generic error analysis method has shown error parameter results are in close agreement with values found with application of binary random numbers, with very large sample size, for all adder architectures with different size, group-size and window-size. Generic analysis method and random probabilistic method based error rate calculation varies by the least, with a value of 0.49% and a maximum of 13.85%.

## Introduction

BEYOND CMOS research the International Roadmap for Devices and Systems (IRDS) includes research in computing, and it adds a number of critical domains and technologies like machine learning, approximate computing and Internet of Things (IoT)^1^. In contrast to the passive use of redundancies like technology scaling, approximate computing employs active design methodologies that exploit the feature that many systems and applications can tolerate some loss of accuracy in the computation result^2^. This error resilience arises from limited human perception, the absence of a golden solution in some algorithms, noise in the input data, and errors in analog-to-digital conversion^3^. The major goal of approximate computing is to design circuits and systems with improved performance by trading off accuracy. Efforts in approximate computing cover a broad spectrum of research, ranging from addressing issues at circuit and system level to the software and application level^4^. Approximate computing works perfectly for error-resilient applications due to aspects like insensitivity to erroneous outputs due to human perceptual limitations. Every system can not be designed to generate correct results, to acceptable level, in the noisy atmosphere, or results with errors are acceptable in error resilient applications. Approximate computing is preferred in real-time applications because it provides faster computations. Errors can be compensated by adding positive or negative offset in the connecting stages of the circuit. Also, in certain applications, inaccurate solutions over a tolerable range are acceptable for a given input^2,5^. There are many applications that can be benefited from approximate computing such as Artificial Intelligence (AI)^6^, machine learning (ML) , data mining, image processing^7^, audio processing, wireless communication, Internet of Things (IoT)^8^ etc.

Approximate computing can be implemented in adders, multipliers etc. Addition is the most basic and widely used operation in almost all data-related applications like signal processing, machine learning, big data applications, data mining, etc. A literature survey reveals several high-performance approximate adders as an alternative to traditional ones to improve their performance^9–18^.

In order to decide the usability of approximate circuits in application, it is necessary to perform analysis in terms of circuit parameters (area, power, delay etc.) and error parameters (Error Rate (ER), Average Error (AE), Acceptance Probability (AP) etc.) for any approximate circuit^19–24^.

The critical path of adders involve carry chain from Least Significant Bit (LSB) of input (usually input carry to addition) to Most Significant Bit (MSB) of addition (usually output carry). Many approximate adders truncate this carry chain in order to reduce delay and use carry prediction/error correction techniques to reduce the error introduced due to carry truncation. The carry prediction/error correction circuits adds extra area and/or power overhead^3^. Approximate adders can be divided broadly into four categories: approximate full adder (at transistor level) based approximate adders, speculative adders, segmented adders, approximate carry-select adders. Our proposed adder is segmented adder which improve the accuracy by combining smaller carry generator block into larger block without loosing parallelism of carry generate operations.

The novelty in this work is as follows :

- The parallelism in the calculation of group generate signals is improved by combining smaller groups by utilizing multiplexers whose select line is driven by group generate signal generated by lower group generate signal. This reduces the error in the addition of inputs without change in the delay in the calculation of sum bits of adder. The adaptive reconfigurable approximate adder can be made delay optimized by connecting input from lower group to fast input of multiplexer.
- The mathematical model is developed for error analysis by iteratively using probability of bit generate, bit propagate and bit kill function. The proposed generic method of error analysis takes care of approximation used at any bit position for any kind of adder. The error parameter calculations based on randomly generated numbers applied to different architectures has shown results in close agreement with error parameter calculation with proposed generic method of error analysis.

In this paper, error parameters are evaluated for 1,000,000 randomly generated inputs. In an attempt to reduce error and to provide configurable circuit that trade with a varying degree of approximation, we provide the following:

- The multiplexer select line is independently set/reset by external signal (ApproxRCON). ApproxRCON signal which is equal to Adder_size/Group_size.
- Parallel computations of carry signals to reduce the delay of the n-bit additions.
- Carry prediction circuit reuses a part of look ahead circuit rather than building extra dedicated prediction circuitry.
- While operating in approximate mode, part of a circuit is getting bypassed which will reduce hardware complexity with improvement in circuit performance parameters.
- An optimized architecture based on the proposed algorithm, testing it for both exact and approximate modes for analyzing errors.

## RAP-CLA architecture

Reconfigurable APproximate Carry Look-ahead Adder (RAP-CLA) builds upon the accurate Carry Look-Ahead (CLA) adder design by incorporating reconfigurable elements. It uses a portion of the CLA circuitry for carry prediction, which helps in reducing overall area compared to a full CLA implementation. RAP-CLA benefits from the accuracy of CLA during normal operation. It utilizes the reconfigurable carry prediction to switch to approximate mode when necessary, thereby balancing performance and power efficiency.

RAP-CLA achieves a smaller area footprint compared to a full CLA adder, yet it typically occupies more area than simpler adder designs like basic CLA due to the added complexity of reconfigurable elements. RAP-CLA manage errors through carry prediction mechanisms eliminating the need for traditional error correction codes and minimizing data stalls. RAP-CLA, while more complex due to its reconfigurable nature, offers a balance between accuracy and area efficiency by leveraging the existing CLA architecture. RAP-CLA is preferred for applications demanding finer control over accuracy and area efficiency^17,18^.

**Fig. 1:**
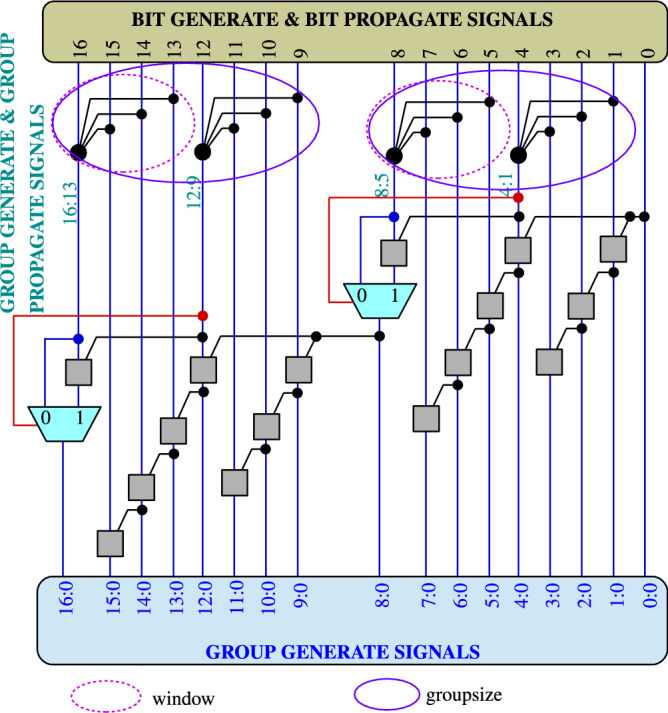
Reconfigurable approximate carry lookahead adder.

**Fig. 2:**
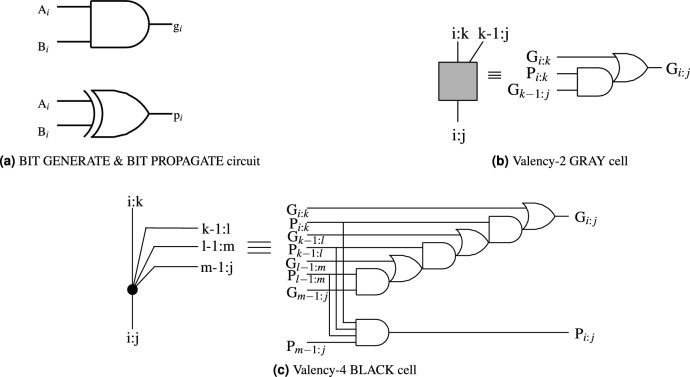
Building blocks of adder architecture.

The structure of the RAP-CLA is based on modifications to the structure of the conventional CLA as shown in Fig. 1. The basic circuits of bit-generate & propagate, Gray Cell and Black Cell are shown in Fig. 2a–c respectively.

The bit generate ($g_i$), bit propagate ($p_i$) and bit kill ($k_i$) functions are defined for each pair of adder inputs ($\hbox {A}_i$, $\hbox {B}_i$), where $\hbox {A}_{N:1}$ and $\hbox {B}_{N:1}$ are two N-bit numbers to be added.

1 $$\begin{aligned} g_i=A_i \cdot B_i, \ \ \ \ \ \ \ \ p_i=A_i \oplus B_i, \ \ \ \ \ \ \ \ k_i=\overline{A_i + B_i} \end{aligned}$$

The group generate signal ($\hbox {G}_{i:j}$) and group propagate signal ($\hbox {P}_{i:j}$) (spanning from bit position *i* to *j*) can be recursively formed by dividing into subgroups as

2 $$\begin{aligned} G_{i:j}=G_{i:k}+P_{i:k}\cdot G_{k-1:j} \end{aligned}$$

and

3 $$\begin{aligned} P_{i:j}=P_{i:k} \cdot P_{k-1:j} \end{aligned}$$

where $i\ge k > j$ with the base case $\hbox {G}_{i:i}$=$g_i$, $\hbox {P}_{i:i}$=$p_i$, $\hbox {G}_{0:0}$=$\hbox {C}_{in}$ (input carry), $\hbox {P}_{0:0}$=0.

The sum and carry bit at position *i* will be $S_i=p_i \oplus G_{i_1:0}$ and $C_i=G_{i:0}$ respectively in exact adders. In approximate adders carry is truncated such that $C_{i}^{approx}=G_{i:j}$ where $i\ge j>0$. The error in carry generation is inversely proportional to size of carry generator block. We use this property in order to improve the error parameters.

RAP-CLA uses CLA as its baseline architecture. In CLA, each bit’s carry-out is computed in parallel by considering the carry- in from lower-order bits, making it efficient for high-speed addition. RAP-CLA optimizes area efficiency by reusing a portion of the CLA circuitry for carry prediction rather than introducing dedicated prediction circuitry. The reconfigurable carry prediction mechanism in RAP-CLA dynamically adjusts based on input conditions or configuration settings, enabling the adder to switch between accurate and approximate modes as needed. As shown in Fig. 1, higher valency black cells are combined with multiplexer to improve groupsize in order to reduce the error. But the delay is not affected as grouping of black cells occurs in parallel across size of the adder. RAP-CLA achieves a practical balance between performance, area efficiency, and flexibility by integrating reconfigurable carry prediction within the CLA framework. This makes it suitable for applications where both speed and adaptability are crucial factors^17,18^.

In RAP-CLA, the carry generation unit is divided into two segments: The first segment covers the most significant terms (MS terms) of the adder, typically denoted by a window size W. The second segment handles the remaining least significant terms (LS terms). This segmentation allows for differential treatment of carry generation based on the significance of the bits within the adder. The MS segment often requires more precise carry handling compared to the LS segment due to their impact on higher-order results.

Initially, all carry generators within the adder may operate under a single mode, typically the approximate mode, for efficiency. To improve accuracy in the approximate mode, especially for MS bits, a technique involves switching the MS segment to use an exact carry generator. This precise handling helps mitigate errors that could propagate significantly through the higher-order bits. The ability to partition the adder into segments with distinct operating mode signals allows for designs with adjustable levels of accuracy. For adders requiring different levels of accuracy or operation under varying constraints (such as power consumption or speed), this partitioning strategy offers a practical approach to optimizing performance without compromising overall functionality. Coordinating the operating mode signals ensures that each segment of the adder operates according to its required accuracy level, thereby enhancing the overall reliability and precision of the computational results. While adding segmentation introduces complexity, the benefits in terms of accuracy improvement and adaptability to diverse operational requirements justify the additional design effort.

This technique is particularly useful in CLA designs where accuracy requirements can vary widely across different parts of the adder. By selectively applying exact carry generation to critical segments (such as MS terms), designers can achieve optimal balance between accuracy and efficiency. The architecture involves gray cells, black cells, and multiplexers to generate ‘Bit Generate’, ‘Bit Propagate’, ‘Group Generate’, and ‘Group Propagate’ signals.

Overall, the segmented carry generation approach in RAP-CLA exemplifies a strategic method to enhance accuracy reconfigurability in adder designs. By partitioning the adder into segments with tailored operating modes, designers can effectively optimize performance characteristics based on specific application needs, ensuring both accuracy and efficiency in computational operations^18^.

RAP-CLA demonstrates higher accuracy compared to GeAr across various metrics such as Error Rate (ER), Mean Relative Error Distance (MRED), and Normalized Error Distance (NED). These metrics indicate that RAP-CLA produces more reliable results in both approximate and exact modes. Increasing the window (segment) size in RAP-CLA enhances all design parameters, further improving its accuracy and performance. Even with larger window sizes, RAP-CLA has higher operating speed compared to the exact CLA with compromise on error parameters.

## Proposed architecture

**Fig. 3:**
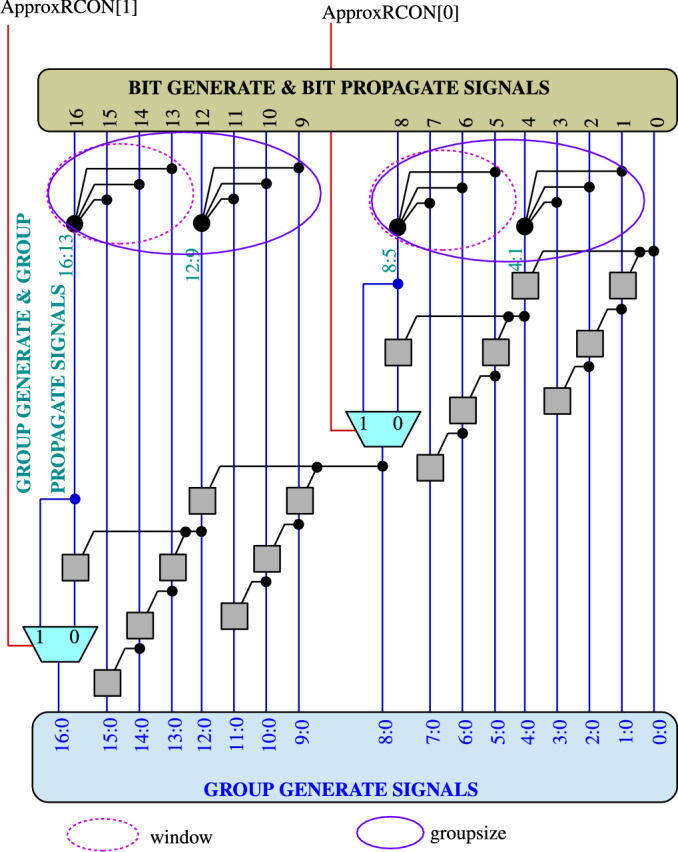
Proposed reconfigurable approximate carry look-ahead adder with exact mode operation.

Proposed approximate adaptive carry-lookahead adder for error resilient applications is flexible to work in approximate as well as exact mode. Proposed architecture differs from previous RAP-CLA design^25^ and shown in Figure 3. The architectural differences are highlighted as:

1. The multiplexer input “1” is fed by output of higher 4 bits valency black cell of individual group and input “0” is fed by gray cell output. This gray cell is fed by output of higher 4 bits valency black cell of individual group and gray cell output connected at highest bit-position in lower group of individual groups. Inputs to this gray cell are one from lower 4 bits valency black cell of individual group and another from output of previous group or $\hbox {C}_{in}$ in case of lowest group.
2. Select line of multiplexer is defined as ApproxRCON and it is controlled by external signal either 0/1. Proposed adder works in exact mode when select line signal set to zero and in approximate mode when ApproxRCON set to 1.

During approximate mode, part of circuit is bypassed [3 bits at LSB side in each group] due to which delay is reduced along with hardware complexity but error rate exceeds at some extent. Also the improvement in delay can be attributed to the connection of slow outputs, especially the group generate signal of black cell to the fast input of multiplexer. The sizing of gates at transistor level is function of fanout of the gate and in our proposed architecture, the lower black cell has to drive only gray cell while upper black cell has to drive gray cell and multiplexer input, thereby improving area overhead in comparison to conventional approximate adders.

In exact mode, RAP-CLA achieves smaller design parameters compared to GeAr, although larger than those of the exact CLA due to additional reconfigurability overhead. RAP-CLA excels in approximate mode as well, leveraging its reconfigurable carry prediction to optimize performance while minimizing error propagation. This mode is particularly beneficial in applications where slight inaccuracies are acceptable in exchange for improved speed and efficiency.

RAP-CLA outperforms GeAr by offering competitive performance in both exact and approximate modes without the extensive delay associated with multicycle error recovery. This flexibility makes RAP-CLA suitable for diverse applications requiring either high precision or faster computational speeds. The ability of RAP-CLA to seamlessly switch between approximate and exact modes enhances its versatility. It caters to applications where dynamic adjustment between accuracy levels is crucial, accommodating both error-resilient tasks and tasks demanding high precision.

In summary, RAP-CLA stands out as a robust solution for configurable adder design, providing superior accuracy, performance efficiency, and versatility compared to GeAr. Its ability to operate effectively in both approximate and exact modes ensures optimal performance across a range of computational tasks and application requirements^18^.

One of the limitation of proposed method is that it can not achieve the performance in terms of delay as that of approximate carry-select adder as this method pre-computes the results for input carry of logic ’0’ as well as logic ’1’ using duplicate blocks and selection of results is made thereafter but with additional area overhead. Segmented Approximate Adder with Smart Chaining (SASC) has comparable error parameters and delay with additional overhead of area^3^.

## Design analysis

In this section, the circuit parameters (Timing, Area, Power—TAP) of different architectures are analyzed. All architectures were written in parameterized verilog code with Size, GroupSize and WindowSize as parameters and are synthesized using Cadence Genus tool using 45 nm technology. All architecture were synthesized with same constraints (setup time, hold time, clock frequency and load capacitor) and same operating conditions.

**Fig. 4:**
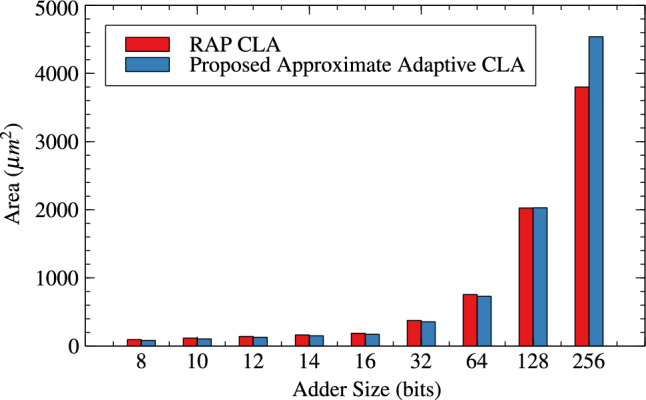
Area comparison for RAP-CLA and AACLA.

**Fig. 5:**
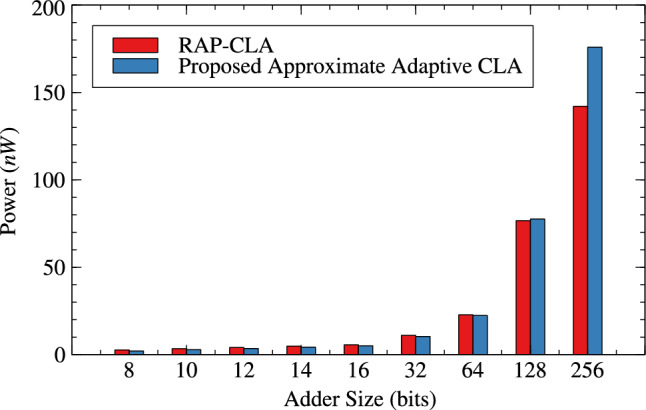
Power comparison for RAP-CLA and AACLA.

**Fig. 6:**
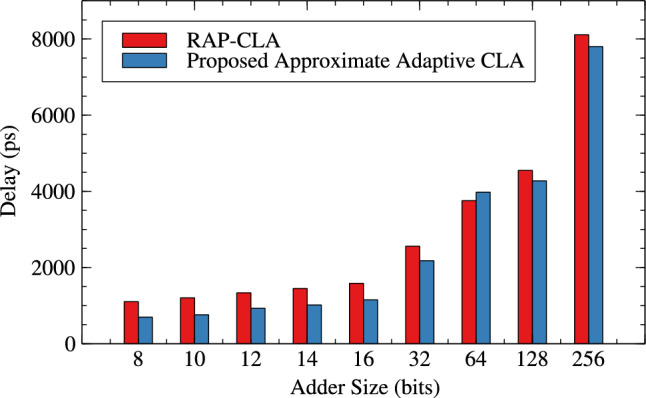
Delay comparison for RAP-CLA and AACLA.

Table 1,2 and Figs. 4, 5 and 6 are respectively about area, power and delay parameter analysis of RAP-CLA and proposed RAP-CLA. After comparing data from Tables 1 and 2, it is observed that the proposed RAP-CLA shows improvement with respect to original RAP-CLA in the following way: In case of 32 bit length, area is optimized by 4.91% while Power is optimized by 5.59%. Delay is reduced by 14.92% over RAP-CLA. The improvement in delay is due to bypassing the first gray cell in each group during the approximate operation and combining of black cells to make larger groups without loosing on parallelism of black cell operations throughout the size of adder. This advantage is of course will not be available in exact mode of operation.

**Table 1:** RAP-CLA TAP reports.

Size	Area ($\upmu m^2$)	Power (W)	Delay (ps)
8	93.708	2.70523$\times 10^{-6}$	1106
10	117.648	3.39647$\times 10^{-6}$	1206
12	141.588	4.07074$\times 10^{-6}$	1336
14	164.16	4.83831$\times ^10{-6}$	1449
16	186.732	5.60121$\times ^10{-6}$	1584
32	375.516	1.10271$\times ^10{-5}$	2559
64	756.162	2.27940$\times ^10{-5}$	3757
128	2025.632	7.66358$\times ^10{-5}$	4549
256	3800.065	1.42064$\times ^10{-4}$	8110

**Table 2:** Proposed RAP-CLA approximate mode TAP reports.

Size	Area ($\upmu m^2$)	Power (W)	Delay (ps)
8	81.396	2.12553$\times ^10{-6}$	697
10	105.336	2.80972$\times ^10{-6}$	759
12	127.908	3.51540$\times ^10{-6}$	930
14	150.480	4.27330$\times ^10{-6}$	1016
16	172.368	5.04047$\times ^10{-6}$	1152
32	357.048	1.04155$\times ^10{-5}$	2177
64	728.460	2.23947$\times ^10{-5}$	3979
128	2027.872	7.75601$\times ^10{-5}$	4275
256	4539.109	1.75913$\times ^10{-4}$	7798

Tables 4 and 5 are about effect of change in group-size and window-size on resource parameters. When group size and window size is reduced to half than original then there is an improvement in all three resource parameters for variable bit lengths. It is observed that as the bit length increases from 16 to 256 the improvement in circuit parameters like area, power and delay are $-0.36$% to 10.42%, 0.71% to 13.20% and, -23.61% to 35.21% for original RAP-CLA. For proposed RAP-CLA with ApproxRCON =1 are 5.55% to 5.99%, 15.11% to 5.93%, 39.49% to 45.07%.

**Fig. 7:**
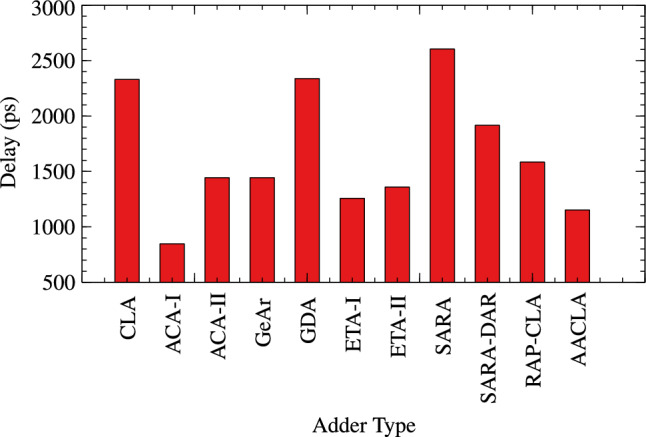
Delay of adders.

**Fig. 8:**
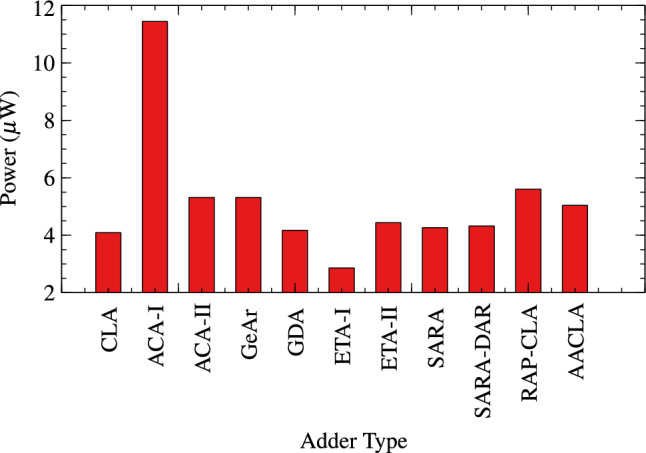
Power dissipation in adders.

**Fig. 9:**
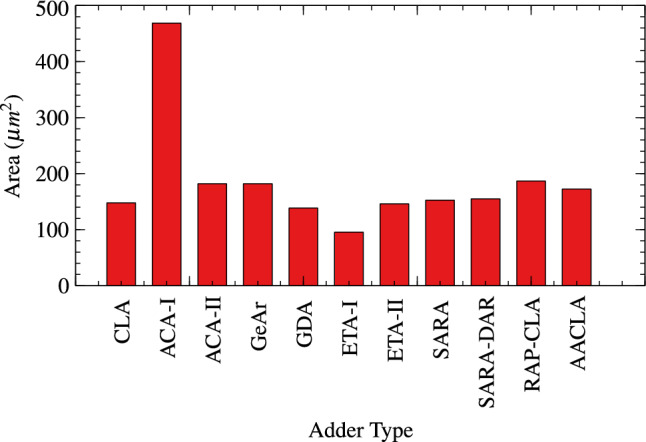
Area in adders.

Proposed RAP-CLA is compared with 9 different approximate adders and one exact adder CLA for TAP analysis. The results are tabulated in Table 3. Bar graph of these parameters are shown in Figs. 7, 8, and 9.

**Table 3:** TAP Analysis of different adder architectures (size 16).

Delay (ps)	Power ($\upmu W$)	Area ($\upmu m^2$)	Architecture	Type of adder
2330	4.089	147.744	CLA	Exact Adder
847	11.44	468.50	ACA-I^26^	Approximate adders
1444	5.311	181.944	ACA-II^26^	
1444	5.311	181.944	GeAr^17^	
2337	4.170	138.510	GDA	
1257	2.8584	95.418	ETA-I^27^	
1359	4.44	146.034	ETA-II^28^	
2604	4.266	152.532	SARA^16^	
1916	4.324	154.926	SARA-DAR	
1584	5.601	186.732	RAP-CLA	
1152	5.040	172.368	Proposed RAP-CLA	

**Table 4:** Effect of group and window size on RAP-CLA.

Size_ GroupSize_ WindowSize	Area ($\mu m^2$)	Power (W)	Delay (ps)
16_8_4	186.732	5.60	1584
16_4_2	187.416	5.56	1958
32_16_8	375.516	11.027	2559
32_8_4	373.464	11.31	2436
64_32_16	756.162	22.79	3757
64_16_8	751.032	22.16	3411
128_64_32	2025.632	76.635	4549
128_32_16	1521.900	48.019	3998
256_128_64	3800.065	142.64	8110
256_64_32	3403.926	123.81	5254

**Table 5:** Effect of group and window size on proposed RAP-CLA.

Size_ GroupSize_ WindowSize	Area ($\upmu m^2$)	Power (W)	Delay (ps)
16_8_4	172.368	5.04	1152
16_4_2	162.792	4.2783	697
32_16_8	357.048	10.41	2177
32_8_4	344.736	10.16	1152
64_32_16	728.460	22.39	3979
64_16_8	714.096	20.928	2177
128_64_32	2027.872	77.56	4275
128_32_16	1456.920	45.55	3985
256_128_64	4539.109	175.913	7798
256_64_32	4267.168	165.48	4283

**Fig. 10:**
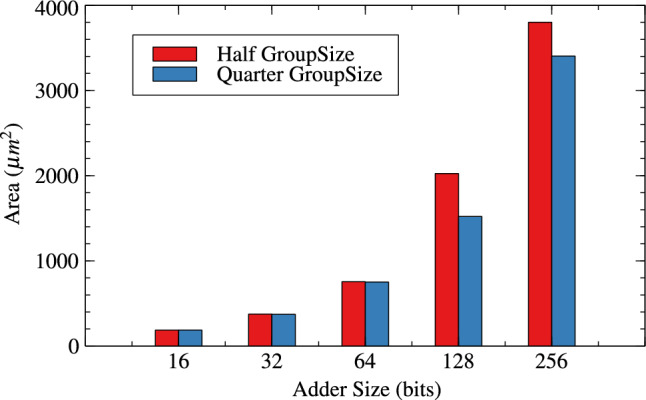
Effect of GroupSize on area for RAP-CLA.

**Fig. 11:**
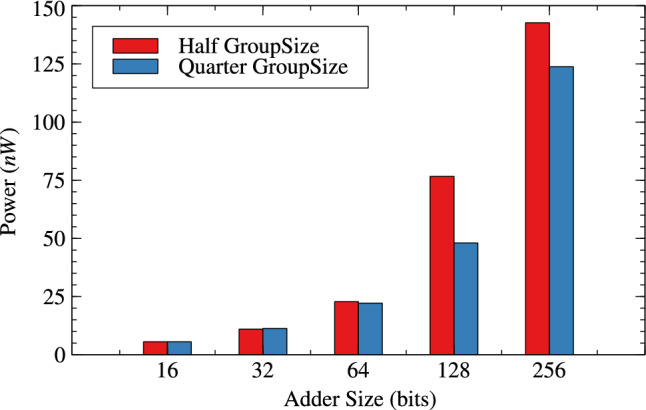
Effect of GroupSize on power for RAP-CLA.

**Fig. 12:**
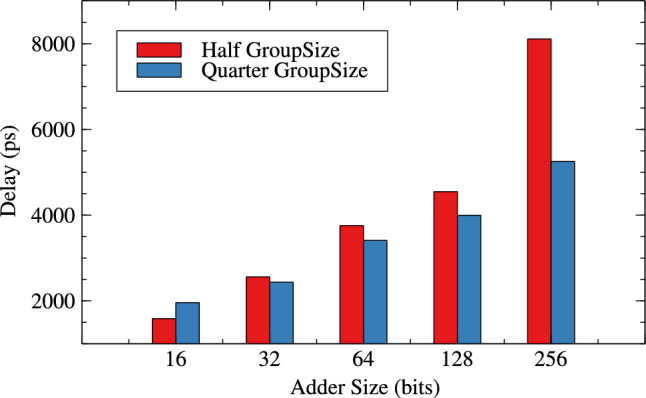
Effect of GroupSize on delay for RAP-CLA.

**Fig. 13:**
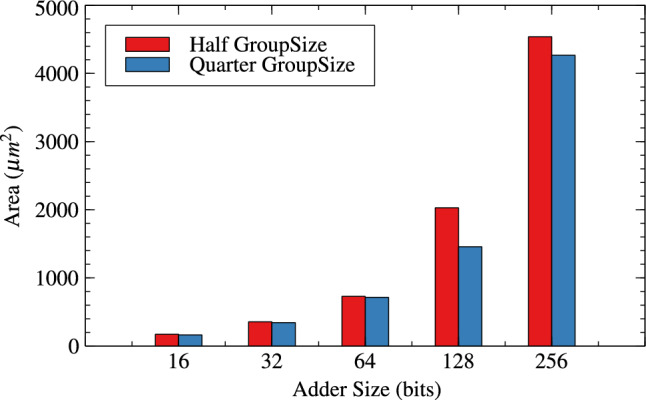
Effect of GroupSize on area for adaptive approximate CLA.

**Fig. 14:**
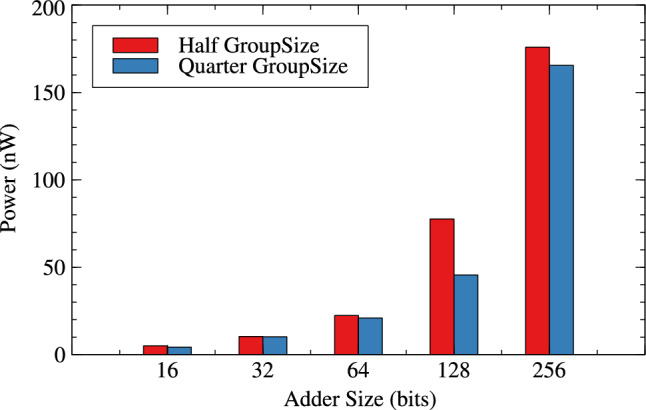
Effect of GroupSize on power for adaptive approximate CLA.

**Fig. 15:**
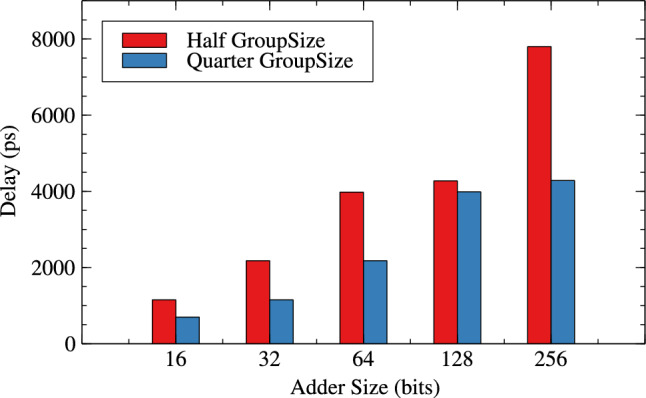
Effect of GroupSize on delay for adaptive approximate CLA.

Figures 10, 11 and 12 shows the effect of group-size on area, power and delay for RAP-CLA while Figs. 13,14 and 15 shows effect on same parameters for proposed adaptive approximate adder.

## Generic method for error analysis

**Fig. 16:**
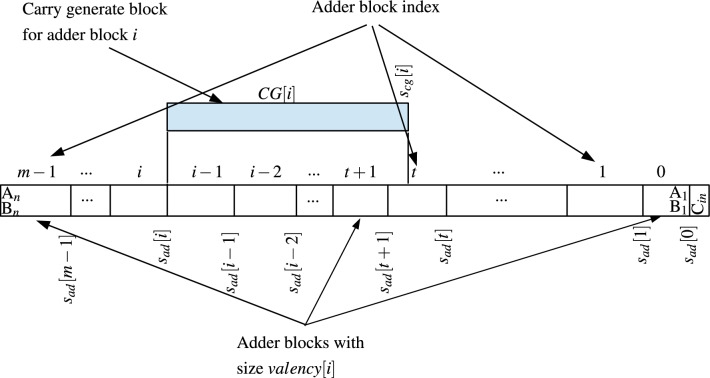
Dividing adder into sub-blocks for error analysis.

Figure 16 shows the terminologies used to develop mathematical model for error analysis. The division of adder in sub-blocks can be of different size and minimum length of sub-block can be 1. The error analysis method takes into consideration the probability of carry kill function. This method can take care of error correction/carry prediction technique, if applied by designer in the approximate adder architecture. Also this method can be used in error analysis of approximate carry-select adders where sum and carry output of blocks are precomputed and results are finally selected through set of multiplexers based on previous carry.

Let the adder of size be *n* bits with two binary inputs $A_nA_{n-1}...A_1$ and $B_nB_{n-1}...B_1$ with $C_{in}$ at $0^{th}$ index. Adder is divided into *m* blocks, each of size *valency*[*i*], where *i* is the index of adder block, $m-1\le i \le 0$. Rightmost block has index 0. We denote bit start index (rightmost bit) of each adder block as $s_{ad}[i]$. Then the leftmost bit index of block will be $s_{ad}[i]+valency[i]-1$.

Let us denote group generate, group propagate and group kill signals of block *i* as *G*[*i*], *P*[*i*] and *K*[*i*] respectively.

4 $$\begin{aligned} G[i]&= G_{(s_{ad}[i]+valency[i]-1):s_{ad}[i]} \nonumber \\&=\sum _{j=s_{ad}[i]}^{s_{ad}[i]+valency[i]-1}g_j\prod _{d=j+1}^{s_{ad}[i]+valency[i]-1}p_d \end{aligned}$$

5 $$\begin{aligned} P[i]&= P_{(s_{ad}[i]+valency[i]-1):s_{ad}[i]} =\prod _{j=s_{ad}[i]}^{s_{ad}[i]+valency[i]-1}p_j \end{aligned}$$

and

6 $$\begin{aligned} \begin{aligned} K[i]&= K_{(s_{ad}[i]+valency[i]-1):s_{ad}[i]}\\ &=\sum _{j=s_{ad}[i]}^{s_{ad}[i]+valency[i]-1}k_j\prod _{d=j+1}^{s_{ad}[i]+valency[i]-1}p_d \end{aligned} \end{aligned}$$

For any one combinations of inputs to the block, only one of the following will be satisfied: $G[i]=1$, $P[i]=1$, $K[i]=1$. Probability of *G*[*i*] and *K*[*i*] to be 1 will be

7 $$\begin{aligned}&P(G[i]=1\ or \ K[i]=1)\\ &=\left( \dfrac{1}{4}\right) _{s_{ad}[i]+valency[i]-1} +\left( \dfrac{1}{2}\cdot \dfrac{1}{4}\right) _{s_{ad}[i]+valency[i]-2}\\ &+ ...+ \left( \dfrac{1}{2}_1\cdot \dfrac{1}{2}_2\cdot ...\cdot \dfrac{1}{2}_{valency[i]-1}\cdot \dfrac{1}{4} \right) _{s_{ad}[i]} \\&=\dfrac{1}{4}\left( \dfrac{1-\left( \dfrac{1}{2}\right) ^{valency[i]}}{1-\dfrac{1}{2}} \right) =\dfrac{1}{2}-\dfrac{1}{2^{valency[i]+1}} \end{aligned}$$

Probability of *P*[*i*] to be 1 will be

8 $$\begin{aligned}&P(P[i]=1)\\ &=\left( \dfrac{1}{2}\right) _{s_{ad}[i]+valency[i]-1} \cdot \left( \dfrac{1}{2}\right) _{s_{ad}[i]+valency[i]-2}\cdot ... \cdot \left( \dfrac{1}{2} \right) _{s_{ad}[i]} \\&=\dfrac{1}{2^{valency[i]}} \end{aligned}$$

Let *CG*[*i*] be the carry generate block for adder block *i* and $s_{cg}[i]$ be the bit start index (rightmost bit index) for *CG*[*i*]. Generally bit end index (leftmost bit index) for *CG*[*i*] will be $s_{ad}[i]-1$.

Let *c*[*i*] be the exact carry in for adder block *i* and $c_{cg}[i]$ be the generated carry in to adder block *i* by its *CG*[*i*] block. If $c[i]=c_{cg}[i]$ for given combinations of input bits, then block *i* will produce sum bits without any error for those input bits. Also for an adder block *i*, if $s_{cg}[i]=0$ for its *CG*[*i*], then $c[i]=c_{cg}[i]$ and block adder *i* will always create exact result.

Let *AR*[*q*] be the event where $c[i]=c_{cg}[i]$ for all $q\le i \le 1$ then the result of addition from block 0 to block *q* will be without error. For an *n* bit approximate adder consisting of *m* adder blocks, if $c[i]=c_{cg}[i]$ for $m-1\le i \le 1$ (i.e. event $AR[m-1]$), the result of addition will be exact (for $i=0$, always $c[i]=c_{cg}[i]=c_{in}$). If probability of this event is $P(AR[m-1])$, then error rate is given by $1-P(AR[m-1])$.

9 $$\begin{aligned} P(AR[0])&={\left\{ \begin{array}{ll} 1 , & \text {if }\ s_{cg}[0]=0\\ 0.5, & \text {if} \ \ s_{cg}[0]=s_{ad}[0]=1 \end{array}\right. } \end{aligned}$$

10 $$\begin{aligned} P(c[1]=c_{cg}[1])&={\left\{ \begin{array}{ll} 1 , & \text {if }\ s_{cg}[1]=0\\ P(K[0])+P(P[0])P(c_{in}=0), & \text {if} \ \ s_{cg}[1]=s_{ad}[1]\\ P(G[0])+P(K[0])+P(P[0])P(c_{in}=0), & \text {if} \ \ s_{cg}[1]=s_{ad}[0]\\ P(G[0_L])+P(K[0_L])+P(P[0_L])P(K[0_R])+\\ \ \ \ \ \ \ \ \ \ \ \ \ \ \ \ \ \ \ \ \ \ \ \ \ \ \ \ \ \ \ \ \ P(P[0])P(c_{in}=0), & \text {if} \ \ s_{ad}[1]> s_{cg}[1] > s_{ad}[0] \end{array}\right. } \end{aligned}$$

11 $$\begin{aligned} P(c[2]=c_{cg}[2])&={\left\{ \begin{array}{ll} 1 , & \text {if }\ s_{cg}[2]=0\\ - - - - - - - - - - - - - - - - - - & - - - - - - - - -\\ P(K[1])+P(P[1])P(K[0])+P(P[1])P(P[0])P(c_{in}=0), & \text {if} \ \ s_{cg}[2]=s_{ad}[2]\\ - - - - - - - - - - - - - - - - - - & - - - - - - - - -\\ P(G[1_L])+P(K[1_L])+P(P[1_L])P(K[1_R])+\\ \ \ \ \ \ \ \ \ \ \ \ \ \ \ \ P(P[1])P(K[0])+ P(P[1])P(P[0])P(c_{in}=0), & \text {if} \ \ s_{ad}[2]> s_{cg}[2]>s_{ad}[1]\\ - - - - - - - - - - - - - - - - - - & - - - - - - - - -\\ P(G[1])+P(K[1])+P(P[1])P(K[0])+\\ \ \ \ \ \ \ \ \ \ \ \ \ \ \ \ \ \ \ \ \ \ \ \ \ \ \ \ \ \ \ \ P(P[1])P(P[0])P(c_{in}=0), & \text {if} \ \ s_{ad}[1]= s_{cg}[2]\\ - - - - - - - - - - - - - - - - - - & - - - - - - - - -\\ P(G[1])+P(K[1])+P(P[1])P(G[0_L])+P(P[1])P(K[0_L])+\\ \ \ \ \ P(P[1])P(P[0_L])P(K[0_R])+P(P[1])P(P[0])P(c_{in}=0), & \text {if} \ \ s_{ad}[1]> s_{cg}[2] > s_{ad}[0] \\ - - - - - - - - - - - - - - - - - - & - - - - - - - - -\\ P(G[1])+P(K[1])+P(P[1])P(G[0])+P(P[1])P(K[0])+\\ \ \ \ \ \ \ \ \ \ \ \ \ \ \ \ \ \ \ \ \ \ \ \ \ \ \ \ \ \ \ \ \ \ \ \ \ P(P[1])P(P[0])P(c_{in}=0), & \text {if} \ \ s_{cg}[2]= s_{ad}[0]\\ - - - - - - - - - - - - - - - - - - & - - - - - - - - -\\ \end{array}\right. } \end{aligned}$$

12 $$\begin{aligned} P(c[3]=c_{cg}[3])&={\left\{ \begin{array}{ll} 1 , & \text {if }\ s_{cg}[3]=0\\ - - - - - - - - - - - - - - - - - - & - - - - - - - - -\\ P(K[2])+P(P[2])P(K[1])+P(P[2])P(P[1])P(K[0])+\\ \ \ \ \ \ \ \ \ \ \ \ \ \ \ \ \ \ \ \ \ \ \ P(P[2])P(P[1])P(P[0])P(c_{in}=0), & \text {if} \ \ s_{cg}[3]=s_{ad}[3]\\ - - - - - - - - - - - - - - - - - - & - - - - - - - - -\\ P(G[2_L])+P(K[2_L])+P(P[2_L])P(K[2_R])+P(P[2])P(K[1])+\\ \ \ \ \ P(P[2])P(P[1])P(K[0])+P(P[2])P(P[1])P(P[0])P(c_{in}=0), & \text {if} \ \ s_{ad}[3]> s_{cg}[3]>s_{ad}[2]\\ - - - - - - - - - - - - - - - - - - & - - - - - - - - -\\ P(G[2])+P(K[2])+P(P[2])P(K[1])+P(P[2])P(P[1])P(K[0])+\\ \ \ \ \ \ \ \ \ \ \ \ \ \ \ \ \ \ \ \ \ \ \ \ \ \ \ \ \ \ P(P[2])P(P[1])P(P[0])P(c_{in}=0), & \text {if} \ \ s_{ad}[2]= s_{cg}[3]\\ - - - - - - - - - - - - - - - - - - & - - - - - - - - -\\ P(G[2])+P(K[2])+P(P[2])P(G[1_L])+P(P[2])P(K[1_L])+\\ \ \ \ \ \ \ \ \ \ \ P(P[2])P(P[1_L])P(K[1_R])+P(P[2])P(P[1])P(K[0])+\\ \ \ \ \ \ \ \ \ \ \ \ \ \ \ \ \ \ \ \ \ \ \ \ \ \ \ \ \ \ \ P(P[2])P(P[1])P(P[0])P(c_{in}=0), & \text {if} \ \ s_{ad}[2]> s_{cg}[3]> s_{ad}[1] \\ - - - - - - - - - - - - - - - - - - & - - - - - - - - -\\ P(G[2])+P(K[2])+P(P[2])P(G[1])+P(P[2])P(K[1])+\\ \ \ \ \ \ \ P(P[2])P(P[1])P(K[0])+P(P[2])P(P[1])P(P[0])P(c_{in}=0), & \text {if} \ \ s_{ad}[1]= s_{cg}[3] \\ - - - - - - - - - - - - - - - - - - & - - - - - - - - -\\ P(G[2])+P(K[2])+P(P[2])P(G[1])+P(P[2])P(K[1])+\\ \ \ \ \ \ \ \ \ \ \ \ P(P[2])P(P[1])P(G[0_L])+P(P[2])P(P[1])P(K[0_L])+\\ \ \ \ \ \ \ \ \ \ \ \ \ \ \ \ \ \ \ \ \ \ \ \ \ \ \ \ \ \ \ \ \ \ \ \ P(P[2])P(P[1])P(P[0_L])P(K[0_R])+\\ \ \ \ \ \ \ \ \ \ \ \ \ \ \ \ \ \ \ \ \ \ \ \ \ \ \ \ \ \ \ \ \ \ \ \ P(P[2])P(P[1])P(P[0])P(c_{in}=0), & \text {if} \ \ s_{ad}[1]> s_{cg}[3] > s_{ad}[0] \\ - - - - - - - - - - - - - - - - - - & - - - - - - - - -\\ P(G[2])+P(K[2])+P(P[2])P(G[1])+P(P[2])P(K[1])+\\ \ \ \ \ \ \ \ \ \ \ \ \ \ P(P[2])P(P[1])P(G[0])+P(P[2])P(P[1])P(K[0])+\\ \ \ \ \ \ \ \ \ \ \ \ \ \ \ \ \ \ \ \ \ \ \ \ \ \ \ \ \ \ \ \ \ \ \ \ \ P(P[2])P(P[1])P(P[0])P(c_{in}=0), & \text {if} \ \ s_{cg}[3]= s_{ad}[0]\\ - - - - - - - - - - - - - - - - - - & - - - - - - - - -\\ \end{array}\right. } \end{aligned}$$

Generalized equation is

13 $$\begin{aligned} P(c[i]=c_{cg}[i])={\left\{ \begin{array}{ll} 1 , & \text {if }\ s_{cg}[i]=0\\ - - - - - - - - - - - - - - - - - - & - - - - - - - - - - - - - -\\ \sum \limits _{m=j}^{i-1} P(G[m])\prod \limits _{n=m+1}^{i-1}P(P[n])+\\ \sum \limits _{m=0}^{i-1}P(K[m]) \prod \limits _{n=m+1}^{i-1}P(P[n])+\\ P(c_{in}=0)\prod \limits _{m=0}^{i-1}P(P[m]), & \text {if} \ \ s_{cg}[i]=s_{ad}[j], \ i \ge j \ge 0\\ - - - - - - - - - - - - - - - - - - & - - - - - - - - - - - - - -\\ \sum \limits _{m=i-t_i+1}^{i-1}\left( P(G[m])+P(K[m]) \right) \prod \limits _{n=m+1}^{i-1}P(P[n])+\\ \left[ P(G[(i-t_i)_L]) + P(K[(i-t_i)_L])\right] \prod \limits _{m=i-t_i+1}^{i-1}P(P[m])+\\ \left[ P(P[(i-t_i)_L])P(K[(i-t_i)_R])\right] \prod \limits _{m=i-t_i+1}^{i-1}P(P[m])+\\ \ \ \ \ \ \ \ \ \ \sum \limits _{m=0}^{i-t_i-1}P(K[m])\prod \limits _{n=m+1}^{i-1}P(P[n])+ & \text {if} \ \ s_{ad}[i-t_i+1]>s_{cg}[i] >s_{ad}[i-t_i],\\ \ \ \ \ \ \ \ \ \ \ \ \ \ \ \ \ P(c_{in}=0)\prod \limits _{m=0}^{i-1}P(P[m]), & i\ge t_i\ge 1\\ - - - - - - - - - - - - - - - - - - & - - - - - - - - - - - - - -\\ \end{array}\right. } \end{aligned}$$

For values of *r* in the limit $i-1\ge r \ge 0$, if $s_{cg}[i] \le s_{ad}[r]$, all such adder blocks with index *r* are sunblocks of *CG*[*i*]. For any adder block of index *t* for which $s_{ad}[t+1]>s_{cg}[i]>s_{ad}[t]$, such adder block is partially part of *CG*[*i*]. We will assume that such adder block with index *t* is virtually divided into two sub-blocks : (1) Right sub-block $t_R$ that is not part of *CG*[*i*] with bit index $s_{cg}[i]-1\ge bit\_index\ge s_{ad}[t]$-Let us denote group generate, group propagate and group kill signals as $G[t_R]$, $P[t_R]$ and $K[t_R]$ respectively; (2) Left sub-block $t_L$ that is part of *CG*[*i*] with bit index $s_{ad}[t+1]-1 \ge bit\_index \ge s_{cg}[i]$-Let us denote group generate, group propagate and group kill signals as $G[t_L]$, $P[t_L]$ and $K[t_L]$ respectively.

The bit-length of *CG*[*i*] is $s_{ad}[i]-s_{cg}[i]$. In order to calculate the number of adder blocks completely covered by *CG*[*i*], following condition should be satisfied by *t*:

14 $$\begin{aligned} \sum _{x=i-t+1}^{i-1}valency[x] \le s_{ad}[i]-s_{cg}[i] < \sum _{x=i-t}^{i-1}valency[x] \end{aligned}$$

Then the number of adder blocks completely covered by *CG*[*i*] will be $i-1-t$. Adder block with index $i-t$ may have been partially covered.

The probability of accurate results is given by

15 $$\begin{aligned} P(AR[m-1])=\prod \limits _{i=0}^{m-1}P(c[i]=c_{cg}[i]) \end{aligned}$$

and the Error Rate (ER) is given by

16 $$\begin{aligned} ER=1-P(AR[m-1]) \end{aligned}$$

## Error analysis result discussion

The mathematical model developed in the previous section is applied to different adder architectures including proposed adaptive approximate adder.

**Fig. 17:**
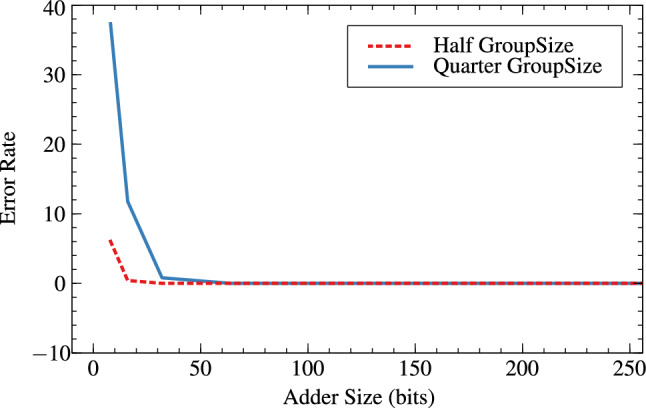
Effect of GroupSize on error rate (ER) for RAP-CLA.

**Fig. 18:**
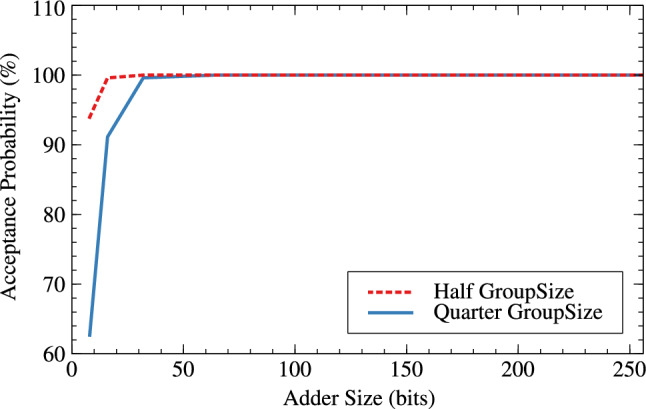
Effect of GroupSize on acceptance probability (AP in %) for RAP-CLA.

**Fig. 19:**
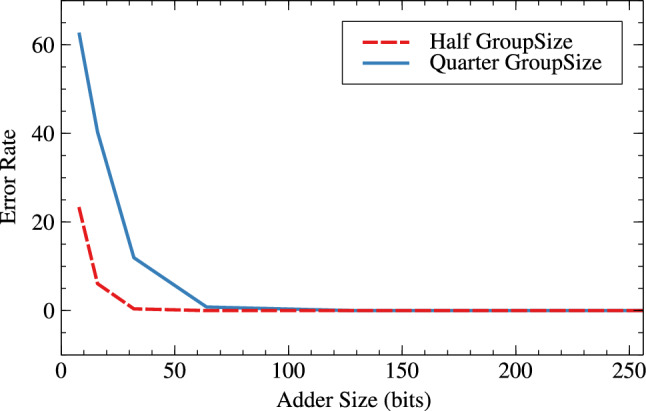
Effect of GroupSize on error rate (ER) for adaptive approximate CLA.

**Fig. 20:**
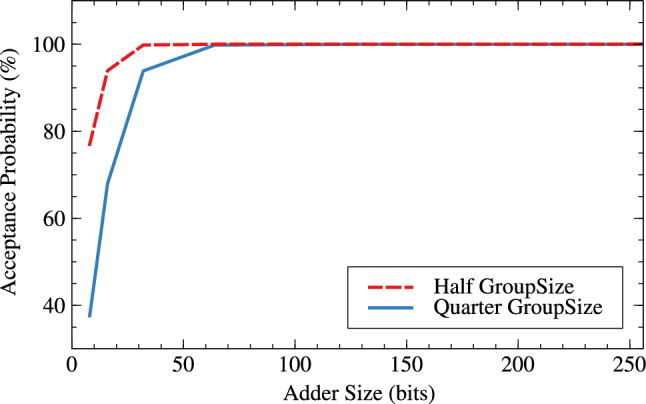
Effect of GroupSize on acceptance probability (AP in %) for adaptive approximate CLA.

Tables 6 and 7 are the error metrics tables for RAP-CLA, proposed RAP-CLA, and effect of group-size and window-size on error parameters in case of both the adders. The error rate is higher in case of proposed RAP-CLA when compared with RAP-CLA. It is about 73.75% for 8 bits and 96.73% for 32 bit lengths. Acceptance probability comparatively reduces from RAP-CLA to proposed RAP-CLA. It reduces with the rate of 18.05% for 8 bits and 0.17% for 32 bits. Tables 6 and 7 also shows effect of group size and window size on error metrics for RAP-CLA (Figs. 17, 18) and proposed RAP-CLA (Figs. 19, 20). When group and window size is reduced then the error rate increases for smaller window and group size. For RAP-CLA, it is found that for 8-bits error rate increased by 83.81% and for 32-bits by 100%. Acceptance probability is reduced by 33.29% and 0.39% for 8 and 32 bits respectively. For proposed RAP-CLA, it is seen that error rate increased by 63.11% for 8-bits and 96.73% for 32- bits. Acceptance probability is reduced by 51.15% for 8 bits and 5.96% for 32 bits.

**Table 6:** Error metrics of RAP-CLA.

Size_GroupSize_WindowSize	Error rate (ER)	Average error (AE)	Acceptance probability (AP)	Hamming distance probability (HDP)	Mean relative error distance (MRED)	Normalized mean error distance (NMED)
8_4_2	6.04	3148.368	93.956	1.109	3.148	3.145
8_2_1	37.32	12422.67	62.678	8.56	12.423	12.516
16_8_4	7.41	2016.883	99.594	0.039	0.217	0.217
16_4_2	11.77	3113.401	91.135	1.308	3.113	3.1
32_16_8	0	1	99.999	0	0.001	0.001
32_8_4	0.8	221.632	99.6	0.043	0.222	0.222
64_32_16	0	0	100	0	0	0
64_16_8	0	3	99.997	0	0.003	0.003
128_64_32	0	0	100	0	0	0
128_32_16	0	0	100	0	0	0
256_128_64	0	0	100	0	0	0
256_64_32	0	0	100	0	0	0

**Table 7:** Error metrics of proposed RAP-CLA.

Size_GroupSize_WindowSize	Error rate (ER)	Average error (AE)	Acceptance probability (AP)	Hamming distance probability (HDP)	Mean relative error distance (MRED)	Normalized mean error distance (NMED)
8_4_2	5.81	2844.63	96.991	1.497	2.845	2.534
8_2_1	32.39	10273.52	37.61	7.168	11.274	10.151
16_8_4	6.08	1315.203	93.92	0.568	3.045	3.104
16_4_2	10.34	1785.12	97.985	5.162	11.785	12.442
32_16_8	0.39	179.752	99.82	0.018	0.18	0.18
32_8_4	0.74	3083.842	93.864	0.677	3.084	3.142
64_32_16	0	0	100	0	0	0
64_16_8	0.8	187.743	99.812	0.022	0.188	0.188
128_64_32	0	0	100	0	0	0
128_32_16	0	1	99.999	0	0.001	0.001
256_128_64	0	0	100	0	0	0
256_64_32	0	0	100	0	0	0

It is clear from Tables 6 and 7 that for higher bit widths (128,256), reduced group and window size affect less on error parameters as compared to smaller window sizes while offering resource parameter improvement. This is the added advantage of the proposed architecture. Table 8 shows the error analysis of different adders using probabilistic method and proposed generic analysis method.

**Table 8:** Comparison of error analysis methods for selected approximate adders.

Type of adder	No. of bits	Error rate using probabilistic method	Error rate using generic method
ACA-I	8_4	1.51	1.5625
	16_12	0.05	0.0488
	32_16	0.02	0.0122
ACA-II	8_2	18.2	18.75
	16_4	5.83	5.859
	32_2	78.32	78.45
ETA-I	8_2_6	91.16	85.16
	8_6_2	71.87	61.91
	16_4_12	98.38	97.75
	16_12_4	84.02	78.9
	32_16_16	99.52	99.33
GeAr	16_2_6	2.591	2.343
	32_2_6	7.08	6.99
RAP-CLA	16_8_4	1.591	1.495
	16_4_2	1.951	1.896
Proposed RAP-CLA	16_8_4	1.522	1.395
	16_4_2	1.643	1.593

The error analysis using randomly generated binary numbers were applied to different adder architectures and error parameters were calculated for very large sample size (1 million for larger adder size). This method is time-consuming and quality of results is function of sample size used in the exercise. However our method of analysis has shown results in close agreement with random number based method.

## Conclusion

The adaptive reconfigurable adder proposed in this paper exploits higher degree of parallelism of Black cell and Gray cells in order to improve the speed and multiplexer used expands the group-size and thus improves the error parameters. The reconfigurability can be used to choose between exact mode and approximate mode for different blocks and hence tune speed and error parameters as per the error resiliency of the application for which it can be used. The proposed approximate adder is designed in such a way that it can inculcate the benefits of exact adder e.g. high acceptance probability, low error rate. Proposed RAP-CLA compared with exact adder CLA for 16 bit total size. Proposed adder is 50.55% time efficient, but consumes 18.86% and 16.66% more power and area. The generic method for error analysis is introduced to provide more flexibility for error calculation. It surpasses random probabilistic method by providing universal algorithm where effect of error prediction/error correction can be included in error analysis along with addition of probability of carry kill function.

The proposed adder can be used in an approximate multiplier for further use in real time applications in domain: Image and signal processing^29^, digital filters^30^, nanotechnology^31^, and IoT^32^ etc.

## Data Availability

The data that support the findings of this study are available from the corresponding author upon reasonable request.
